# Comparison of Efficacy of Combination of Bromelain, Rutocide, and Trypsin With Serratiopeptidase on Postoperative Sequelae Following Mandibular Third Molar Surgery: A Randomized Clinical Trial

**DOI:** 10.7759/cureus.48633

**Published:** 2023-11-10

**Authors:** Raparthi Bhuvan Chandra, Kathiravan Selvarasu, Murugesan Krishnan

**Affiliations:** 1 Oral and Maxillofacial Surgery, Saveetha Dental College and Hospitals, Saveetha Institute of Medical and Technical Sciences, Saveetha University, Chennai, IND

**Keywords:** pain, third molar surgery, serratiopeptidase, bromelain, proteolytic enzymes

## Abstract

Introduction

Pain, swelling, trismus, and dry socket are common postoperative complications of mandibular third molar surgery (MTMS), which is a routine minor oral surgical procedure. The purpose of this study was to compare the efficacy of the combination of bromelain, rutocide, and trypsin versus serratiopeptidase in reducing postoperative sequelae after MTMS.

Materials and methods

It was a pilot study conducted from October 2022 to May 2023 in the outpatient department of a private dental institution. Patients with horizontal position C mandibular third molar impaction were enrolled, and a surgical procedure was performed. Patients were divided into two groups. Group A received tab Rutocide D (trypsin 48mg, bromelain 90mg, rutoside 100mg, and diclofenac 50mg) twice daily, and Group B received tab Zerodol SP (aceclofenac 100mg, paracetamol 325mg, and serratiopeptidase 15mg) twice daily in the postoperative period of five days. Outcome parameters like pain, using the visual analog scale (VAS), and mouth opening, were measured on postoperative days one and seven. An Excel spreadsheet (Microsoft, Redmond, WA, USA) was used for data entry and statistical analysis was performed using SPSS (version 23.0; IBM Corp., Armonk, NY, USA). The patients included in the study were analyzed postoperatively. The statistical significance was set at p < 0.05. An independent sample t-test was used to assess the variables between groups.

Results

The data analyzed showed that the pain perception in Group A was less when compared to Group B. On postoperative day one, the mean VAS score in Group A was 4.0 ± 0.3 and in Group B was 5.2 ± 0.4. On postoperative day seven the mean VAS score in Group A was 1.4 ± 0.43 and in Group B was 3.0 ± 0.4. The results were statistically significant with less pain experienced by participants in Group A compared to participants in Group B on both postoperative day one and postoperative day seven. On the first postoperative day, patients in Group A had a mean mouth opening of 33.68 ± 1.42, whereas patients in Group B had a mean mouth opening of 29.41 ± 1.86, which was statistically significant. Similarly, on postoperative day seven, patients in Group B had a mean mouth opening of 31.73 ± 3.27, whereas patients in Group A had a mean mouth opening of 36.32 ± 0.24, which was statistically significant.

Conclusion

It is concluded that the proteolytic enzyme combination of trypsin, bromelain, and rutocide is more efficacious than serratiopeptidase in reducing postoperative sequelae after MTMS.

## Introduction

Third molar tooth extraction is the most common chairside minor oral surgical procedure performed in clinical practice [1,2]. As with any other surgical procedure, this one is also associated with complications as it involves nerve blocks, incisions, bone guttering, and closure with foreign material. Complications can range from minor (pain, swelling, and limited mouth opening) to severe (alveolar osteitis and lingual nerve paresthesia) [3-5]. When compared to healthy individuals, patients undergoing impacted third molar surgery have compromised quality of life due to postoperative sequelae such as pain, swelling, and trismus [6-8]. Hence, pain reduction protocols are needed in this kind of surgery.

The inflammatory pathway is governed by prostaglandins, which are produced in the arachidonic acid pathway. Non-steroidal anti-inflammatory drugs reduce pain by inhibiting this pathway [9,10]. Along with glucocorticoids, the role of natural products like bromelain, trypsin, and papain is being given importance [11,12]. The pineapple-derived proteolytic enzyme bromelain, which is a botanical member of the Bromlileaceae family, minimizes the inflammatory effects after the impacted third molar surgery [12]. Apart from having anti-inflammatory properties, bromelain also has antiemetic, anti-tumor, and immunomodulatory effects. Bromelain prevents the metabolization of arachidonic acid by inhibiting bradykinin production in the bloodstream via proteolytic degradation of immune complexes. It improves the absorption of antibiotics applied to the skin during burn debridement. It is also used as an adjunct to anticancer chemotherapy drugs. Trypsin is endogenously produced inside the human gut as a proteolytic enzyme that helps in the digestion of proteins. Rutocide is a natural flavin derivative that has anti-inflammatory properties [12-15].

Serratiopeptidase is another proteolytic enzyme that is derived from silkworms. A microorganism called Serratia E15, which inhabits the gut, produces this drug. This suppresses the inflammatory response by causing proteolysis of all devitalized tissues, such as blood clots, tissue plaques, and cellular debris. Because it does not involve the prostaglandin pathway, it is considered safe for patients with gastrointestinal problems [16-18]. The aim of this study was to evaluate the efficacy of a combination of bromelain, trypsin, and rutocide with serratiopeptidase on postoperative sequelae after impacted mandibular third molar surgery (MTMS).

## Materials and methods

Study design and setting

This clinical trial was commenced in the outpatient department of Oral and Maxillofacial Dentistry at a private dental institution in Chennai for a period of eight months. The trial was registered in the clinical trial registry of India (REF/2023/09/073649) and the study was conducted after obtaining ethical clearance from the institution (IHEC/SDC/OMFS-2101/23/166). 

Study population 

Patients with impacted mandibular third molar, horizontal, position C, and class III on radiographic and clinical examination were included in the study. The allocation was done using an opaque envelope. The patients were randomly divided into two groups in a 1:1 ratio, as follows: bromelain, rutocide, and trypsin (Rutocide D) were given to Group A (n = 22). Only serratiopeptidase was given to Group B (n = 22). Patients between the ages of 20 and 40, both genders, and with a radiographic diagnosis of a horizontal, position C, or class III impacted tooth were eligible [19]. Inclusion criteria included patients who were willing to adhere to the study protocol, patients who had had no oral surgical interventions in the previous three weeks, and patients who were free of pain and other inflammatory symptoms such as swelling and decreased mouth opening at the time of surgery. Pregnant and lactating women and patients with medical comorbidities were excluded from the study. The proforma contained information about individual cases. Panoramic radiographs were taken for all patients. The treatment procedures were explained to the patients in their native language and written consent was obtained from all the patients.

Intervention 

**Figure 1:**
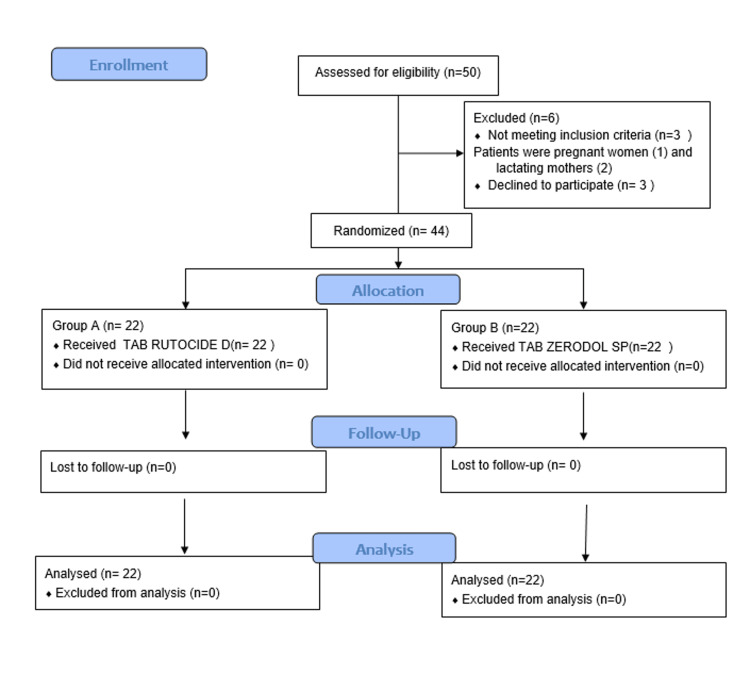
Consolidated Standards of Reporting Trials (CONSORT) flow diagram

The trial comprised 44 patients in total with a p ≤ 0.05 and 95 power, the sample size was calculated using the G* power calculation 3.1.2 software (Heinrich-Heine-Universität Düsseldorf, Düsseldorf, Germany) based on the research done by Murugesan et al. in 2012 [13]. Patients who underwent mandibular third molar surgery were included in the study and were randomly into two groups. Group A (n = 22) and Group B (n = 22) were assigned depending on the analgesic used postoperatively. After surgery, Group B received tab Zerodol SP while Group A received tab Rutoside D. All of the surgical procedures were carried out by a single operator. The patients were allocated into two groups based on sealed opaque envelopes prepared by the investigator and both the operator and the participant were unaware of the study grouping (double blinding). The mandibular anaesthesia was induced with 2% lignocaine hydrochloride with 1:100,000 epinephrine using an inferior alveolar nerve block. Full thickness mucoperiosteal flap was elevated and the tooth was elevated and removed. Closure was done using 3-0 silk. In Group A, postoperative tab Rutoside D (trypsin (48mg) + bromelain (90mg) + rutocide (100mg) + diclofenac (50mg)) was given twice daily for five consecutive days from the day of surgery and in Group B, tab Zerodol SP (aceclofenac (100mg) + paracetamol (325mg) + serratiopeptidase (15mg)) was given twice daily postoperative for five days from the day of surgery. Figure 1 shows the Consolidated Standards of Reporting Trials (CONSORT) flow diagram of the present study.

Follow-up and assessment 

Postoperative clinical assessment and comparative analysis were performed among Group A and Group B patients on postoperative day one and postoperative day seven for pain using visual analogue scale (VAS) score and mouth opening (in millimeters). The pain was assessed using a VAS score from 0 to 10 on day one and day seven post-surgical removal of the impacted tooth, wherein a score of 0 = no pain, values between 0-3 = mild pain, values 4-7 = moderate pain, values between 8-10 = severe pain, and a score of 10 = most severe pain. Pain intensity at various time intervals was compared among the two groups. In both groups, postoperatively patients were prescribed antibiotics for five days and 0.12% chlorhexidine gluconate mouthwash for a week. 

Statistical evaluation

The statistical data was analyzed using SPSS for Windows version 23.0 (IBM Corp., Armonk, NY, USA). All data were summarized as mean ± SD for continuous variables, numbers, and percentages for categorical variables. The comparative statistical test adopted to compare pain scores and maximal mouth opening (in mm) between the two groups was the Independent samples t-test. A p-value of <0.05 was considered to be statistically significant.

## Results

**Figure 2:**
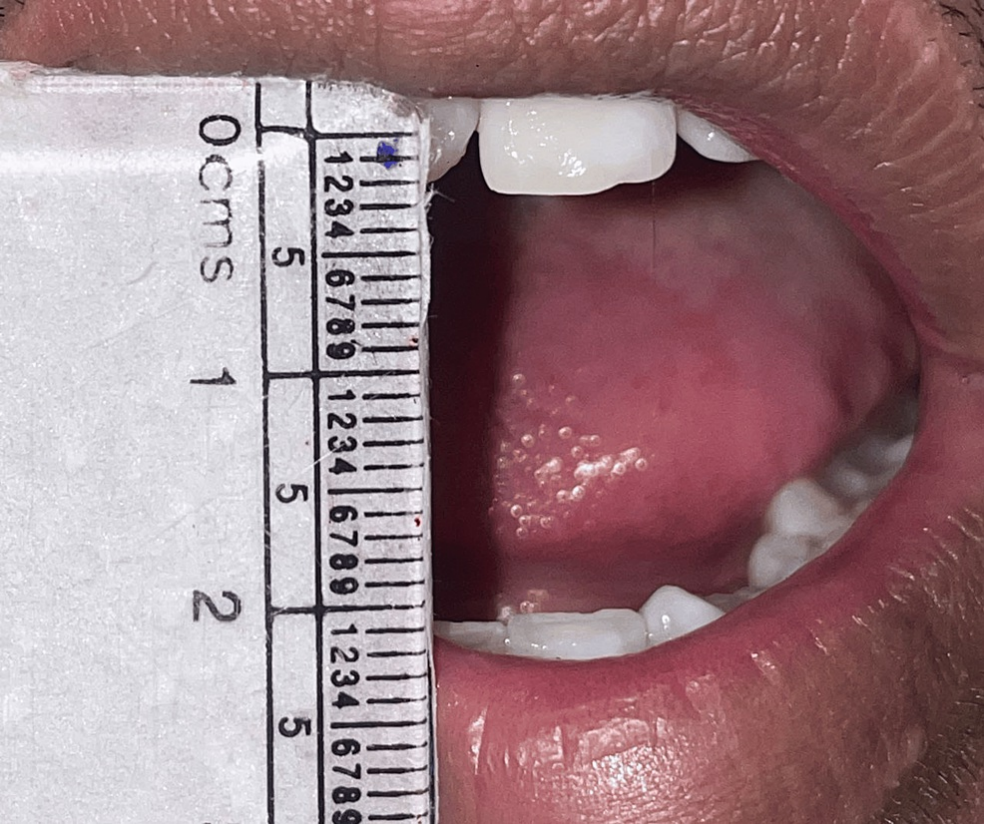
Measurement of mouth opening on postoperative day one

**Figure 3:**
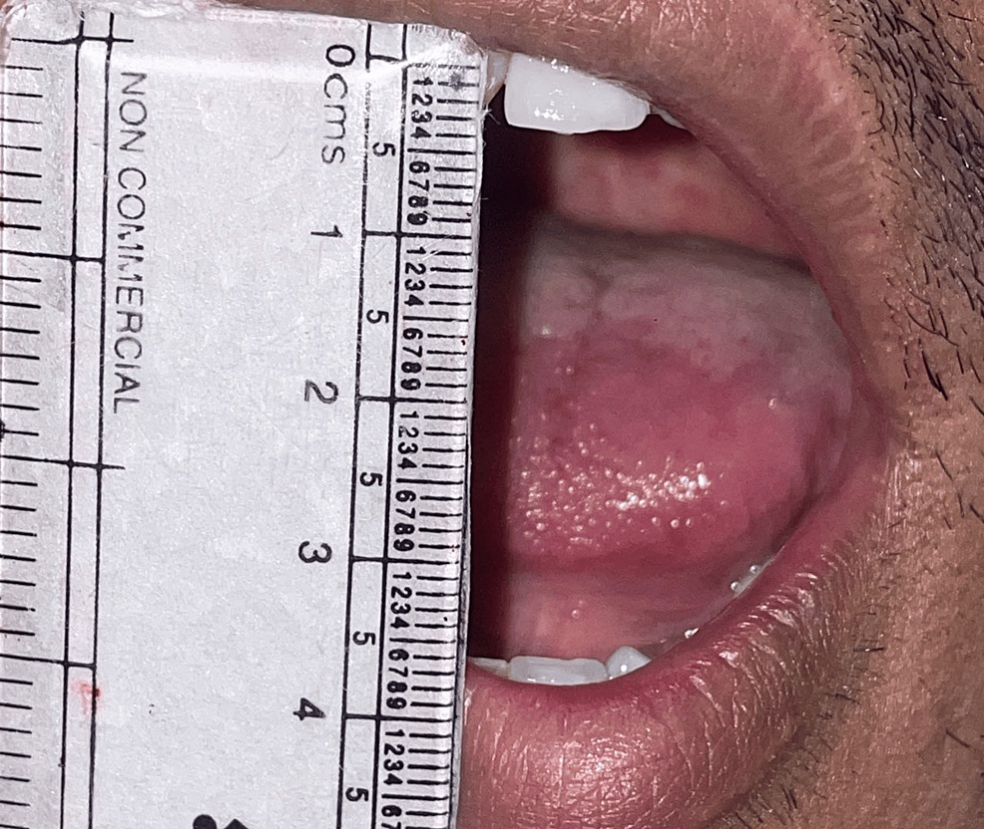
Measurement of mouth opening on postoperative day seven

Mouth opening was measured on postoperative day one (Figure 2) and postoperative day seven (Figure 3) by taking into account the interincisal distance.

The participants of the study were segregated into two groups with 22 in each. All the patients were similar in their socio-democratic characteristics; thus, steps have been taken to minimize the confounding factors (Table 1). 

**Table 1:** Demographic data of the study participants (expressed in mean and standard deviation)

DEMOGRAPHIC DATA	GROUP A	GROUP B
Age (years)	28 ± 1.9	25 ± 1.3
Gender (Male, Female)	12, 10	14, 8
Weight (kg)	66 ± 2.8	64 ± 1.5
Duration of surgery (min)	32 ± 5.2	27 ± 3.6

**Figure 4:**
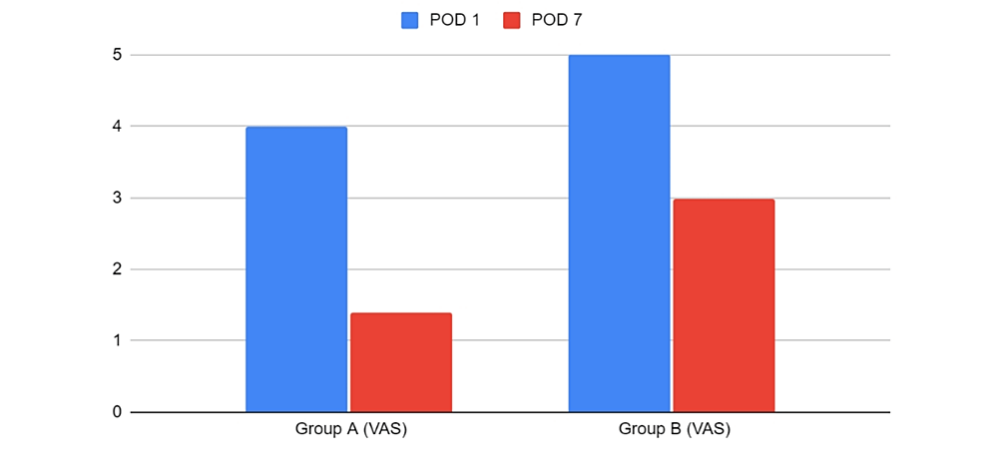
Pain perception among the two study groups VAS - Visual analogue scale, POD - Postoperative day

The mean VAS scores of groups A and B are shown in Figure 4. On postoperative day one, the mean VAS score in Group A was 4.0 ± 0.3 and in Group B was 5.2 ± 0.4. On postoperative day seven the mean VAS score in Group A was 1.4 ± 0.43 and in Group B was 3.0 ± 0.4. The results were statistically significant (p=0.001) with less pain experienced by participants in Group A compared to participants in Group B on both postoperative day one and postoperative day seven.

The mouth openings of patients on postoperative day one and postoperative day seven were compared using an independent sample t-test. On the first postoperative day, patients in Group A had a mean mouth opening of 33.68 ± 1.42, whereas patients in Group B had a mean mouth opening of 29.41 ± 1.86 and the results were statistically significant (Table 2). Similarly, on postoperative day seven, patients in Group B had a mean mouth opening of 31.73 ± 3.27, whereas patients in Group A had a mean mouth opening of 36.32 ± 0.24 and the results were statistically significant. Thus, mean mouth opening was higher in Group A compared to Group B on both postoperative days one and seven.

**Table 2:** Mouth opening on postoperative day one and day seven among the two study groups * - statistically significant at p<0.05, SD - standard deviation, N - number

Independent t-test		N	Mean (SD)	F value	p-value*
Mouth opening on postoperative day 1	Group A	22	33.68 (1.42)	0.692	0.000
	Group B	22	29.41 (1.86)		
Mouth opening at postoperative day 7	Group A	22	36.32 (0.24)	0.338	0.000
	Group A	22	31.73 (3.27)		

## Discussion

Being the most common minor oral surgical procedure, impacted third molar surgery is associated with sequelae like pain, swelling and trismus [10]. To reduce complications, steroids and analgesics are prescribed. We can reduce a few postoperative complications by administering nonsteroidal anti-inflammatory drugs (NSAIDs) prior to surgery. These nonsteroidal anti-inflammatory medications interfere with arachidonic acid metabolism. As the NSAIDs are absorbed rapidly and are distributed in the tissues before the surgical insult happens, they are given as preemptive analgesia [13]. As a result, prostaglandin synthesis is reduced prior to initiating any trauma and, as a result, inflammatory events. There will be a significant decrease in pain and swelling if we reduce the complications of inflammation prior to trauma [14].

Steroids are also used to reduce swelling following surgery and trismus. According to a study, facial swelling was compared between a dexamethasone group and a proteolytic enzyme group. They concluded that facial swelling was less in the steroid group when compared to the proteolytic enzyme group [14]. In terms of trismus, there was no statistically significant difference between groups. The dexamethasone group had significantly lower mean pain and swelling scores than the controls in the previous studies conducted to determine the anti-inflammatory effects of dexamethasone. 

In addition to steroids and NSAIDs, proteolytic enzymes are used to reduce postoperative complications. The most commonly used proteolytic enzymes are trypsin, bromelain, rutocide, and serratiopeptidase [14]. A randomized control trial to compare methylprednisolone and serratiopeptidase in reducing postoperative swelling discovered that swelling outcomes were statistically indistinguishable between groups 1 and 2 on postoperative days one and three, and statistical significance was observed on postoperative day five [15]. Thus, serratiopeptidase is more effective than methylprednisolone in controlling postoperative edema. When comparing groups 1 and 2, the results were statistically significant. Trismus improvement was greater in Group 2 than in Group 1. As a result, serratiopeptidase outperforms methylprednisolone in treating trismus as shown in several studies [16-18].

The study by Tachibana et al. [6] evaluated the efficacy of bromelain in relieving swelling and pain after having third molars surgically removed, and it concluded that 28 patients (70%) had reduced swelling and pain. This study compared the effects of proteolytic enzymes on postoperative sequelae after wisdom tooth extraction. In terms of pain, there was a significant difference between the bromelain and serratiopeptidase groups (P<0.05).

On day one, the average pain rating in Group I was significantly lower than that in Group II. This finding indicates that the addition of bromelain improves pain control after surgery on day one. On day two, the mean pain score in Group I was significantly lower than that in Group II. This finding indicates that patients who received bromelain had better control over postoperative pain than the other groups and the results were in accordance with other similar studies [19-24].

Limitations of the study

A limitation of our study is the small sample size. Further multi-centric studies with larger sample sizes would provide important information about the clinical effectiveness of the drugs used in our study. 

## Conclusions

It is concluded that the proteolytic enzyme combination of trypsin, bromelain, and rutocide is more efficacious than serratiopeptidase in reducing postoperative sequelae after MTMS. This study also enabled us to understand that proteolytic enzymes are helpful in reducing the inflammatory response when combined with routine analgesics in the management of postoperative sequelae after MTMS thereby increasing the quality of life of the patient. 
